# Coping with Heat: Function of The Natal Coat of Cape Fur Seal (*Arctocephalus Pusillus Pusillus*) Pups in Maintaining Core Body Temperature

**DOI:** 10.1371/journal.pone.0072081

**Published:** 2013-08-08

**Authors:** Nicola Erdsack, Guido Dehnhardt, Wolf Hanke

**Affiliations:** Institute for Biosciences, Sensory and Cognitive Ecology, University of Rostock, Rostock, Germany; Texas A&M University-Corpus Christi, United States of America

## Abstract

Cape fur seal (*Arctocephalus pusillus*) pups spend the first weeks of life exclusively or mainly ashore. They are exposed to intense solar radiation and high temperatures for long time periods, which results in temperatures up to at least 80°C on their black natal coat. To test the hypothesis that the natal coat has a crucial function in coping with these extreme conditions, we investigated the insulating properties of the natal coat in six captive newborn Cape fur seals during the first 50 days after birth. The natal fur differs from the adult fur not only in colour, but also in density, structure, and water repellence. We measured temperature on the fur surface and within the fur, as well as skin and rectal temperature under varying environmental conditions, comparable to the species' habitat. Experiments were designed to not influence the spontaneous behaviour of the pups. Rectal temperature was constant as long as the pups stayed dry, even during long-lasting intense solar radiation for up to 3 h. Skin temperature remained close to rectal temperature as long as the fur was dry, while with wet fur, skin temperature was significantly reduced as well. Our results show that the natal coat provides an effective insulation against overheating. The severely reduced insulation of wet natal fur against cold supports the assumption that the natal fur is an adaptation to the pups' terrestrial phase of life.

## Introduction

Due to their global distribution, pinnipeds have to face diverse thermoregulatory requirements imposed by their environment. Phocid seals, highly adapted to the aquatic environment, rely on their blubber as thermal insulation, while their fur is generally of minor thermoregulatory relevance [1], [2], [3], [4] due to low hair density and the lack of underwool [5], [6]. The more terrestrial fur seals contrarily are insulated by a dense water repellent fur. For the pups of seal species distributed in the polar and sub polar regions, particularly ice breeding species, the greatest demand is the prevention of heat loss during the first days of life. The pups of the spotted seal (*Phoca largha* Pallas, 1811) and the harp seal (*Pagophilus groenlandicus* Erxleben, 1777), for example, are born with a light coloured natal fur, the lanugo, that protects them against the cold until they have developed an adequate blubber layer, as long as they stay ashore and dry [7], [8]. The pups of the harp seal additionally have thermogenic brown adipose tissue (BAT) [9], where fat is oxidized with intense heat production instead of ATP synthesis [10]. By contrast, harbour seal (*Phoca vitulina* Linnaeus, 1758) pups shed the lanugo in utero [7] and are born with a blubber layer and an adult-type fur, since they have to be able to swim shortly after birth.

For the pups of species distributed in moderate and tropical climate zones, such as many otariids, protection against heat absorption is presumably as essential as protection against heat loss. Sea lion and fur seal pups are born with a black or dark brown natal coat which they start moulting not before the sixth week [11]. Like the lanugo of phocids, the otariid natal fur lacks the water-repellent properties of the adult fur, so that the pups drench to the skin while staying in the water or in the rain [12], [13], [14]. Newborn fur seal pups indeed are able to keep afloat right after birth but they are not really capable of swimming [13] before the fifth week ([15] and own observations).

The breeding areas of the South African or Cape fur seal (*Arctocephalus pusillus pusillus* Schreber, 1776) range from Algoa Bay, South East Africa, southwards to the Cape of Good Hope and north westwards to the north coast of Namibia. This distribution comprises a huge climate range from humid with moderate temperatures, e. g. at Bird Island, Algoa Bay at the south-east coast of South Africa [16] up to arid desert climate at the north-west coast of South Africa and the south and north coasts of Namibia. Most breeding sites are on rocky islands or cliff coasts, but some sandy beaches at the Namibian coast are occupied by fur seals as well. Due to the poor swimming abilities of fur seal pups, most pupping sites are not suitable for them to enter the water, so that the pups have to spend the first weeks on shore. Furthermore shaded space is rare at most breeding sites, so that the pups are exposed to intense solar radiation for many hours. As it is rarely possible for the pups to cool down behaviourally by entering the water [17] or drenching their fur [18] like adults, other heat protection mechanisms are required. Adult California sea lions (*Zalophus californianus* (Lesson, 1828)), although less insulated than other otariids [19], become hyperthermic after about 100 min at air temperatures ≥30°C, even without physical activity [20]. Though Cape fur seals, in contrast to *Z. californianus*, possess functioning sweat glands in the naked skin areas of the flippers [21], it is not known how functional these glands are in newborns. Even if they were functional, intensive sweating would easily lead to dehydration since the mothers leave their pups as soon as a few days after parturition for foraging trips of up to ten days [22], [23]. So how do fur seal pups cope with long periods of exposure to intense solar radiation and high ambient temperatures without the opportunity to cool down? One option would be for the pups to live with an increased core body temperature during their first postnatal weeks, as observed e. g. in Southern elephant seal (*Mirounga leonina* (Linnaeus, 1758)) pups [24]. This seems rather unlikely considering that Limberger et al. [25] found constant normal core body temperatures in the pups of the Galapagos fur seal (*Arctocephalus galapagoensis* (Heller, 1904)), which are exposed to a hot and dry environment close to the equator, but with the opportunity to take shelter from solar radiation in natural caves. Here we set up and supported an alternative hypothesis, that is, as long as the pups stay on land, their natal fur acts as such an effective thermal barrier against heat transfer that neither skin nor core body temperature are affected. To test this hypothesis, we investigated six Cape fur seal pups, born and kept in Zoo Rostock, Germany, within their first fifty days after birth under various environmental conditions that they also face in nature. We measured rectal temperature with a veterinary thermometer and the temperature of the skin and the air temperature inside the fur using a mantle thermocouple. The temperature on the outer surface of the fur was measured by infrared thermography (IRT). We tested for correlations between these measured temperatures as well as possible influences of other parameters like air temperature, time of day, age and sex of the pups.

## Materials and Methods

### Experimental animals

A total of six Cape fur seal pups were investigated in Zoo Rostock, Germany, in three consecutive years from 2010 to 2012, one male and one female pup each year, aged between 2 and 50 days. The pups were born between end of May and middle of June within a group of four adult seals (one male and three females), all of them trained and used to working with humans. Rostock is located in the north of Germany at the Baltic Sea coast with moderate climate and average air temperatures of 20°C in the summer months, comparable to the climate during breeding season of several breeding areas at South Africa's south coast [16]. Maximal temperatures, however, are well above 30°C. The fur seals were kept in a wind-sheltered enclosure mainly exposed to solar radiation with only little shaded space, but with access to shallow water. Access to the fur seals was provided by Zoo Rostock.

### Temperature measurements

The pups' surface temperature (T_surface_) was measured with an infrared (IR) thermocamera (Fluke Ti25, Eindhoven, The Netherlands) with an adjusted emissivity of 0.98, being the value for bare skin and dry fur [26]. A digital quick response thermometer (GTH 1170, Greisinger electronic GmbH, Regenstauf, Germany) with an attached mantle thermocouple (TKAL 05030, mawi-therm GmbH, Monheim, Germany; mantle diameter: 0.5 mm, nominal length: 30 mm) was used to measure the air temperature within the fur (herein named “fur temperature” T_fur_), and the skin temperature (T_skin_). To measure T_fur_, the thermocouple was inserted into the fur, parallel to the skin, approximately halfway (see “Accuracy of position of the temperature measurements within the fur”) between skin and fur surface. For measurements of T_skin_ the tip of the thermocouple was placed perpendicularly onto the pups' skin. Since the use of ingested temperature transmitters in newborn, exclusively lactated pups is hardly possible without disturbing the animal, we measured rectal temperature as an index of core body temperature (T_body_). Measurements were conducted by means of a veterinary thermometer (microlife VT 1831, Widnau, Switzerland), inserted 9 cm into the rectum. This position is approximately in the centre of the trunk (torso), due to the pups' small body size at birth and slow growth rate. Air temperature (T_air_) was measured with the digital quick response thermometer (see above) with an attached bead thermocouple probe (80PK-1, Fluke, Eindhoven, The Netherlands).

### Experimental procedure

**Figure 1:**
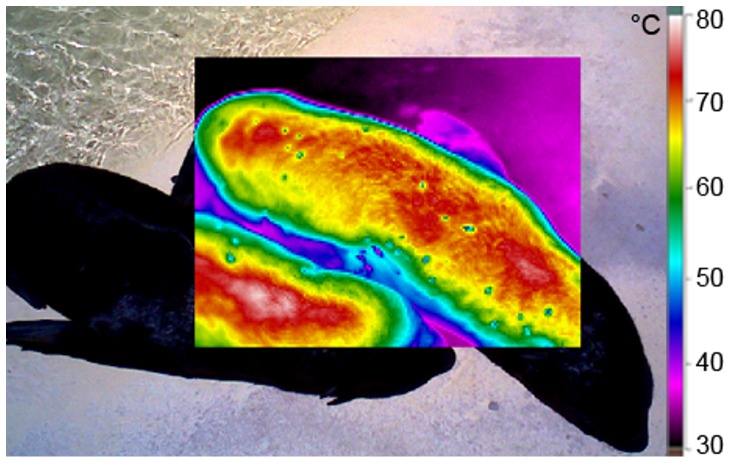
Cape fur seal pups hauling out under intense solar radiation. The pups, aged 18 and 28 days, hauled out in the sun for 1.5 h, T_air_  = 31.3°C. T_surface_ increased up to 78.7°C on their backs.

All temperature measurements were carried out between 9:25 a.m. and 8:10 p.m. All measurements were conducted while the pups were asleep. Measurements started as soon as the pups had hauled out for at least 15 min and were left alone by their mothers. Temperatures were taken at different body parts, in most cases on the dorsal side (neck, back, lower back and head), rarely on the ventral side (abdomen and throat), since the pups mostly hauled out lying on their stomachs (see Fig. 1). T_surface_, T_fur_ and T_skin_ were measured in immediate succession. T_body_ was measured during REM phases of sleep, with the anal sphincter muscle relaxed, to avoid any disturbances of the pups by capturing or waking them up. REM phases could be easily detected by movements of eyes and vibrissae as well as twitching of body parts or the entire body. Additional measurements of T_body_ were made when the pups were caught by the seal keepers for medical examinations. Environmental conditions such as T_air_, cloudiness and precipitation were documented during measurements. Fur condition (dry/wet) and the time the pups had spent on shore prior to the measurement were documented as well.

The experiments were carried out in accordance with the European Communities Council Directive of 24 November 1986 (86/609/EEC). According to § 8 of the German Animal Welfare Act of 18 May 2006 (BGB l. I S. 1206, 1313), experiments conducted in this study were not subject to approval or notification, since they did not cause pain, suffering or injuries to the animals.

### Test for the influence of reflected sunlight on the measurements

We tested if the temperatures measured on the pups' fur surfaces under direct solar radiation are real or potentially involved reflected sunlight measured by the IRT camera. For this purpose we recorded thermograms of a hauled out pup as soon as it was exposed to intense solar radiation until surface temperatures >60°C were measured. Then we shaded a part of the pup's back from the sunlight with a board, took consecutive thermograms and measured the decrease of surface temperature over time.

### Accuracy of position of the temperature measurements within the fur

To estimate the precision of the halfway depth within the fur where T_fur_ had been measured, we carried out 50 control measurements. These measurements were conducted within a piece of fur from the back of a dead born pup. The experimenter who had performed the measurements with the live animals inserted the thermocouple approximately halfway into the fur following the same procedure as during temperature measurements. The depth of the fur as well as the distance between skin and thermocouple were determined by means of a small saw blade as a scale that was inserted perpendicularly through the fur onto the skin. Saw tooth width was 1.1 mm. The tip of the thermocouple was hooked into a kerf of the saw blade. After removing the saw blade with the hooked thermocouple, the distance between skin and thermocouple could be measured. The seal pup skin was provided by Zoo Rostock.

### Data analysis

Thermograms were edited and analyzed with Smartview 3.2 software (Fluke, Eindhoven, The Netherlands). Statistical analyses were carried out with Microsoft Excel 2003 and SPSS 20.0. Data were tested for normal distribution using a chi^2^-test. Equality of variances was tested using an *f*-test. Statistical dependence between values is calculated using Spearman's rank correlation coefficient (r_s_) and its probability of deviating from zero by chance (*p*). Statistical significance of differences of mean values was tested using one-tailed or two-tailed *t*-tests for normally distributed data (core body temperature), a Mann-Whitney *U*-test was used to test for significance in not normally distributed data with equal variance (skin temperature) and a Kolmogorov-Smirnov-test for not normally distributed data with unequal variance (fur surface temperature). Significance level in all cases was α = 0.05.

## Results

A total of 334 measurements of T_surface_, 309 measurements of T_fur_, 307 measurements of T_skin_ and 33 measurements of T_body_ were carried out. T_surface_ increased up to 79.6°C under intense solar radiation. An example thermogram with high temperatures is shown in Fig. 1. Minimum, maximum and mean values along with temperature range and standard deviation for T_surface_, T_fur_, T_skin_ and T_body_ under dry fur and wet fur conditions are given in Table 1. T_air_ varied from 14.4°C to 35.5°C during the study periods, while, apart from few exceptions (see “Impact of wind induced convection”), the air was still (wind speeds below 0.19 m/s). Cloud amount varied from cloudless, with intense solar radiation, to completely overcast.

**Table 1:** T_body_, T_skin_, T_fur_ and T_surface_ of the pups with wet and dry fur.

Temperature		T_min_ (°C)	T_max_ (°C)	range (°C)	T_mean_ ± sd (°C)		N
Total	Body core	36.1	37.7	1.6	36.9	±0.3	33
	Skin	31.5	42.1	10.6	38.1	±1.9	307
	Inside fur	22.7	63.3	40.6	40.0	±7.1	309
	Surface	18.6	79.6	61.0	45.9	±.0	334
Dry fur	Body core	36.1	37.7	1.6	36.9	±0.3	29
	Skin	34.9	42.1	7.2	38.4	±1.7	279
	Inside fur	22.7	63.3	40.6	40.3	±7.1	300
	Surface	19.1	79.6	60.5	47.2	±16.5	316
Wet fur	Body core	36.6	36.9	0.3	36.8	±0.1	4
	Skin	31.5	38.6	7.1	35.8	±1.3	28
	Inside fur	29.3	36.1	6.8	32.5	±2.0	9
	Surface	18.6	29.2	10.6	23.5	±3.0	18

Minimum (T_min_), maximum (T_max_) and mean (T_mean_) temperatures of the pups' fur surface, air inside the fur, skin and body core along with temperature range, standard deviation (s.d.) and number of measurements (N). Total includes the data of all investigated pups with dry and wet fur. Beneath the data of all pups are sorted by wet or dry fur conditions.

### Natal fur structure

The natal fur consisted mainly of coarse bristly hairs of up to 3 cm length. In dry state the hairs were steeply erected and individually curved, thereby forming dorsally and laterally a fur layer of up to about 25 mm depth with an uneven furrowed fur surface. The sparse under hair fibres were much shorter with about 5 mm length, very thin and hardly detectable with the naked eye. Fur depth was largest on neck and head. Hairs on the ventral side were less curved and shorter, thereby forming a thinner fur layer of 5 to 8 mm depth.

### Rectal temperature (all conditions)

**Figure 2:**
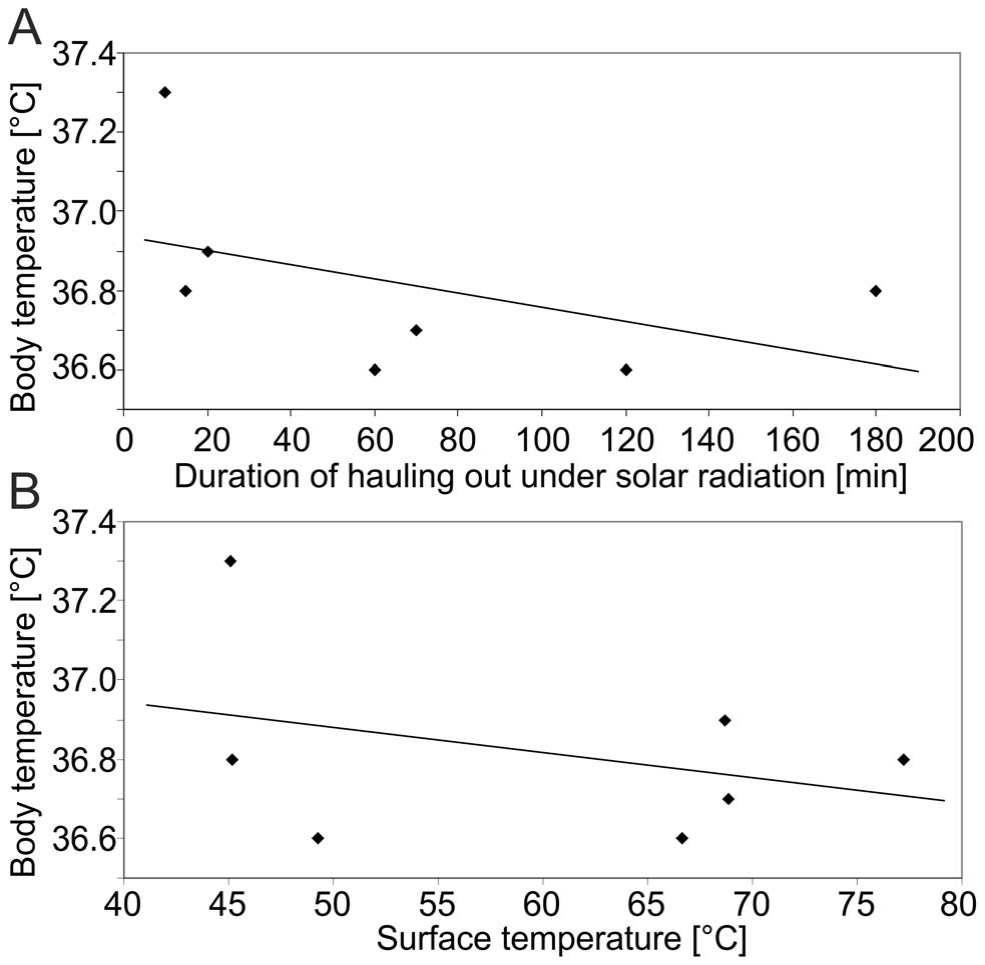
Course of the pups' core body temperature under intense solar radiation. T_body_ of the pups as function of duration of exposure to intense solar radiation (A) and as function of T_surface_ (B). Neither hauling out under intense solar radiation for up to 3 h nor high T_surface_ up to 77.2°C caused an increase of T_body_. Linear regressions have very slightly negative slopes of −0.002 (A) and −0.006 (B) indicating practically constant values.

**Figure 3:**
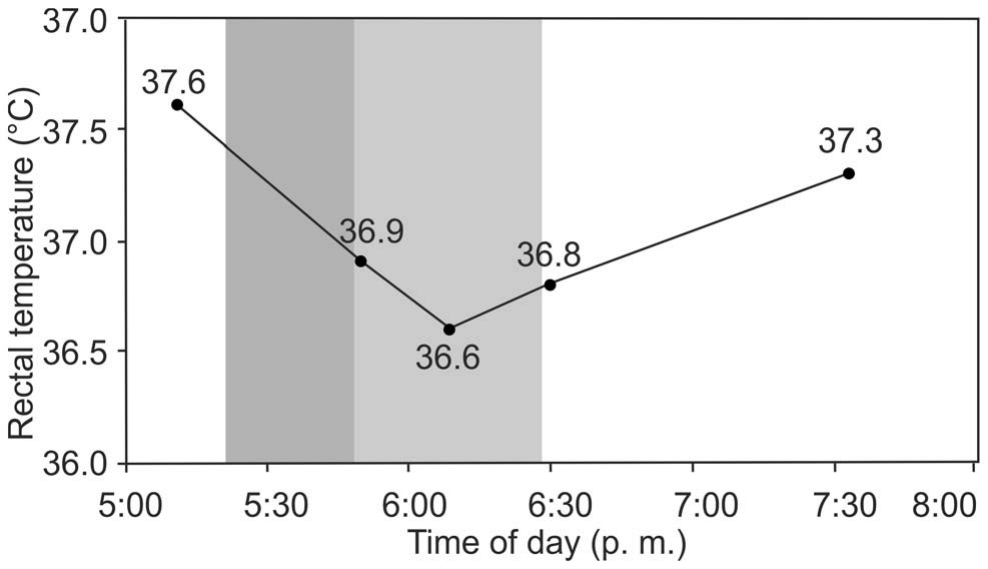
Course of T_body_ of a female pup before, during and after a thunderstorm. The shaded areas indicate the time span with strong rain during the thunderstorm (dark grey) and light rain after the thunderstorm was over (light grey). **5:11 p.m.**: T_air_ = 26.4°C, T_body_ = 37.6°C. Pup had been sleeping in the sun for 1.5 hours. **5:20 p.m.**: A thunderstorm with strong rain started and lasted for 28 minutes. The pup stayed asleep. **5:48 p.m.**: The thunderstorm stopped but it still rained on. **5:50 p.m.**: T_air_ = 19.7°C. T_body_ had decreased by 0.7°C to 36.9°C. **6:09 p.m.**: It was still raining, the pup was still sleeping. T_body_ had further decreased by 0.3°C to 36.6°C. **6:25 p.m.**: T_air_ = 20.4°C. The rain had stopped. **6:30 p.m.**: T_body_ had increased by 0.2°C to 36.8°C. **7:30 p.m.**: T_body_ had further increased by 0.5°C to 37.3°C.

T_body_ was stable throughout the study periods with a mean value of 36.9±0.3°C (mean ±s.d.; N = 33) and was neither significantly correlated to T_skin_ (r_s_ = −0.14; *p* = 0.54), T_fur_ (r_s_ = −0.16; *p* = 0.46), T_surface_ (r_s_ = −0.22; *p* = 0.32), nor to the duration for which the pups were exposed to intense solar radiation (r_s_ = −0.60; *p* = 0.15). Fig. 2 shows the seven values of T_body_ measured under intense solar radiation (10 to 180 min) as a function of the duration of solar radiation (A) and of T_surface_ (B) along with linear regressions. The regression lines have slopes of (A) −0.002 and (B) −0.006, which indicate practically constant values. T_body_ with dry fur measured later than 5:00 p.m. was slightly higher than before 5:00 p.m., at the threshold of statistical significance (36.9±0.3°C before 5:00 p.m.; 37.1±0.4°C after 5:00 p.m.; two-tailed *t*-test with equal variances: *p* = 0.05), while T_body_ with dry and wet fur pooled was not (two-tailed *t*-test with equal variances: *p* = 0.13). No correlations between T_body_ and age of the pups (2 to 50 days of age, r_s_ = −0.08; *p* = 0.68) or T_air_ (14.4°C to 35.5°C, r_s_ = 0.09; *p* = 0.63) were found. T_body_ did not differ significantly between sexes (male: 36.9±0.4°C; female: 36.9±0.3°C; two-tailed *t*-test, equal variances: *p* = 0.71) nor between dry and wet condition of the fur (one-tailed *t*-test, equal variances: *p* = 0.16). However, a set of five successive measurements of T_body_ of a female pup before, during and after a thunderstorm with strong rain features temperature variations of 1.0°C. Data are presented in Fig. 3. The curve shows a decrease of T_body_ by 1.0°C within one hour during the thunderstorm and an increase of 0.7°C within 85 min after the thunderstorm was over.

### Dry fur

When the fur was dry, we measured T_surface_ up to 79.6°C. The test for an impact of reflected sunlight on the measured surface temperatures by shading the animal (see “Test for the influence of reflected sunlight on the measurements”) resulted in a mean decrease of T_surface_ of 0.7±0.5°C per second. This result proved that the measured values were not caused by reflected sunlight, as otherwise temperature decrease in the shade would have been immediate.

The quantification of the location of the measurements of T_fur_ (see “Materials and methods”) resulted in a mean deviation from the centre of fur depth of −0.07±1.19 mm (mean ±s.d., N = 50), showing that the experimenter was able to estimate the halfway point very accurately and supporting the assumption that fur temperatures had been measured halfway between skin and surface.

**Figure 4:**
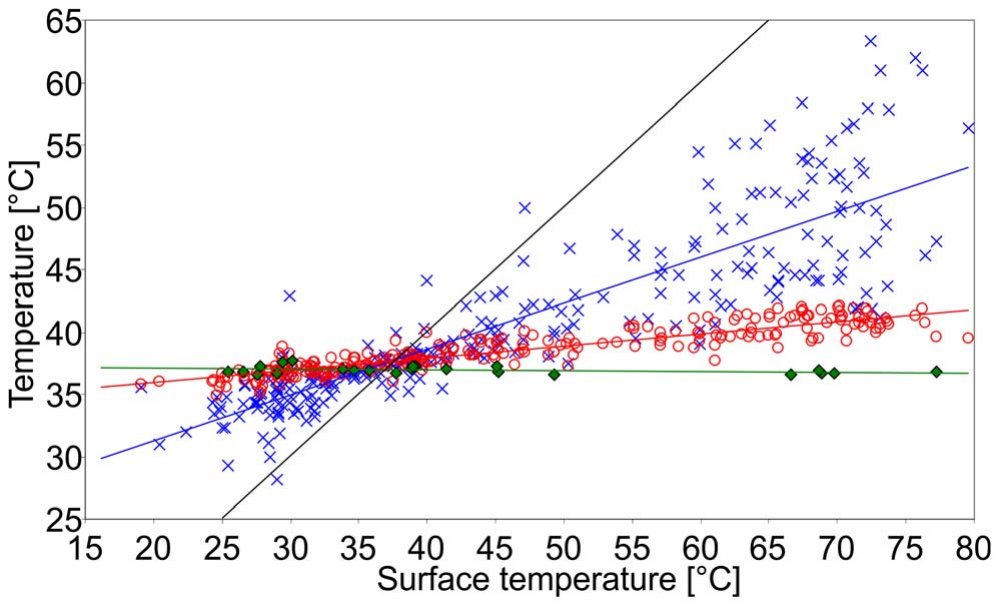
T_body_, T_skin and_ T_fur_ of the pups with dry fur. T_body_ (green diamonds, green regression line; N = 23), T_skin_ (red circles, red regression line; N = 279) and T_fur_ (blue crosses, blue regression line; N = 300) against fur T_surface_ (black line) of the pups with dry fur. At T_surface_ >40°C, T_skin_ and T_fur_ were lower than T_surface_. At T_surface_ <35°C, T_skin_ and T_fur_ were higher than T_surface_. T_body_ was constant at around 37°C (36.9±0.3°C) with a slightly negative slope of regression of −0.006. T_skin_ and T_fur_ have slopes of regression of 0.1 (T_skin_) and 0.4 (T_fur_) and are strongly correlated to T_surface_ (*p*≤0.0001; T_skin_: r_s_ = 0.91; T_fur_: r_s_ = 0.93), while T_body_ is weakly, but not significantly negatively correlated to T_surface_ (r_s_ = −0.22; *p* = 0.32).

In Fig. 4 T_fur_, T_skin_ and T_body_ of the pups with dry fur are plotted as functions of T_surface_ along with linear regressions. Detailed data are presented in Table 1. T_surface_, T_fur_ and T_skin_ were significantly correlated to T_air_ (*p*≤0.0001; T_surface_: r_s_ = 0.64; T_fur_: r_s_ = 0.60; T_skin_: r_s_ = 0.66). T_fur_ and T_skin_ furthermore strongly correlate with T_surface_ (*p*≤0.0001; T_fur_: r_s_ = 0.93; T_skin_: r_s_ = 0.91), while the correlation between T_skin_ and duration of hauling out in the sun was not significant (r_s_ = 0.64; *p* = 0.12). The regression of T_body_ has a slightly negative slope of −0.006, indicating practically constant values, while the slope of regression of T_skin_ is 0.1 and of T_fur_ 0.4. The temperature differences between skin and surface (−40.1°C to +16.7°C; −7.2±14.3°C), skin and fur (−22.6 K to +8.7°C; −2.2±5.6°C) and fur and surface (−32.0°C to 16.5°C; −4.9±10.6°C) were strongly negatively correlated to T_surface_ (skin-surface: r_s_ = −1.0; skin-fur: r_s_ = −0.88; fur-surface: r_s_ = −0.96), all of them highly significantly (*p*≤0.0001).

### Wet fur

**Figure 5:**
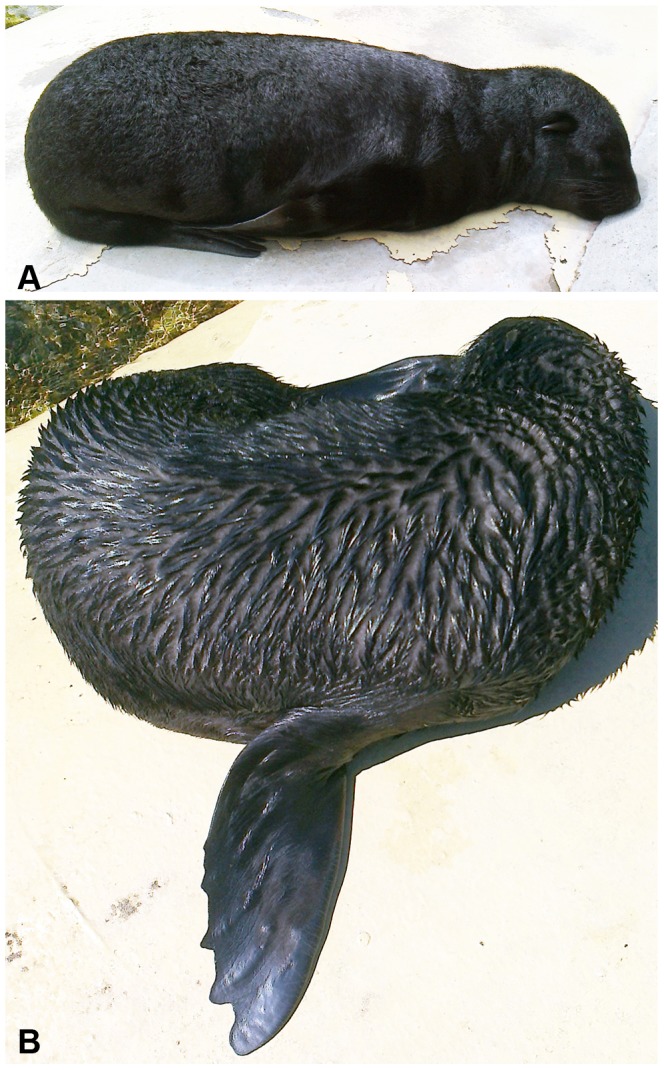
Fur seal pups hauling out with dry and wet fur. (A) Pup with dry fur. The hairs are curled, but constitute a continuous surface. (B) Pup with wet fur, 5 min after leaving the water. The guard hair is divided into strands, opening gaps through which the light grey under fur is visible.

After leaving the water, the pups were drenched to the skin and shook the water out of their pelts like dogs. Fig. 5 shows the different appearances of the dry (A) and the wet fur (B). The wet hair laid down flat and was divided into strands, opening gaps of up to 1 cm width, where the under fur was uncovered. This was also the case when the fur was wetted by rain. On rainy or overcast days with high humidity, the pups' fur did not dry at all. The pups shivered and folded hind and fore flippers under their bodies. They did not sleep as deeply as they did in dry, warm weather, waking up immediately at every touch or contact with the measuring devices.

**Figure 6:**
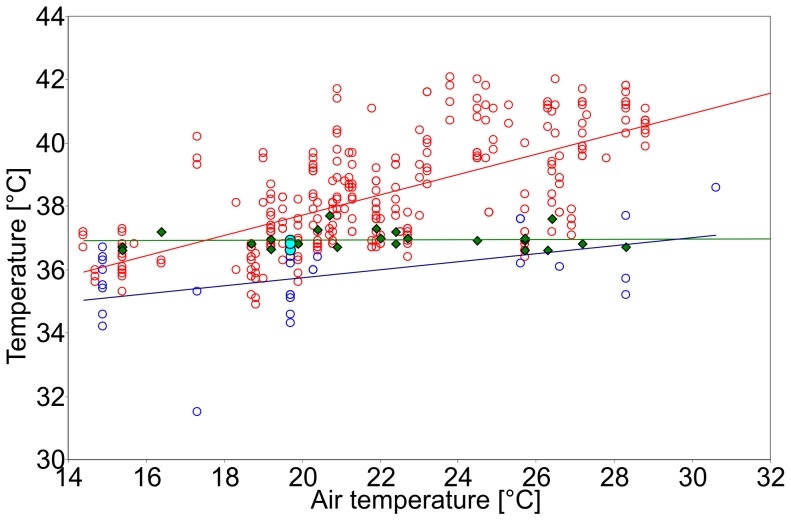
T_body_ and T_skin_ of the pups with wet and dry fur. T_body_ with dry fur (green diamonds, green regression line, N = 23) and wet fur (turquoise filled circles, N = 3) as well as T_skin_ with dry fur (red circles, red regression line, N = 279) and wet fur (blue circles, blue regression line, N = 28) against T_air_. Mean T_skin_ with dry fur is 38.4±1.7°C, mean T_skin_ with wet fur is 35.8±1.3°C, which is significantly lower than with dry fur (*p*≤0.0001). T_body_ is constant with no significant difference between wet and dry fur. The slope of regression of T_body_ is with 0.002 again practically constant, while the regressions of T_skin_ ascend by 0.32 (dry fur) and 0.13 (wet fur).

Detailed data of the temperature measurements with wet fur conditions are presented in Table 1. Due to the flat and stringy condition described above, in most cases measuring of T_fur_ was not possible when the fur was wet, therefore it was excluded from statistical analysis. T_surface_ and T_skin_ were significantly lower with wet fur than with dry fur (Kolmogorov-Smirnov-Test, Mann-Whitney *U*-Test: *p*≤0.0001). The mean value of the nine measurements of T_fur_ in a wet state showed a distinct trend of being lower than the mean value of dry T_fur_ as well. In Fig. 6 T_skin_ and T_body_ with dry and wet fur are plotted as functions of T_air_ along with linear regression lines. T_body_ is again practically constant (slope of the regression line 0.002), while the regressions of T_skin_ have slopes of 0.32 (dry fur) and 0.13 (wet fur).

### Further observations

In the first 10 to 14 days of life three of the pups looked for sheltered places such as gaps in a stone wall, where at least their heads fitted in. During this time the pups were taken into the water by their mothers occasionally. In one case the mother took her pup into the water a few hours after birth, most likely fleeing from the male harassing her. Two pups entered shallow water already from the 3^rd^ week and started swimming in deeper water two weeks later. The other four pups started going into the water between the 4^th^ to 5^th^ week, starting to swim not before the 6^th^ week. From the 5^th^ week on all pups showed active thermoregulatory behaviour on very warm sunny days. They hauled out at the edge of the pool and either submerged shortly in the shallow water every 10 to 15 min, or hauled out with their fore or hind flippers in the water. Panting, licking or sweating, which would be conceivable behavioural mechanisms of heat dissipation, were not observed in any of the pups.

**Figure 7:**
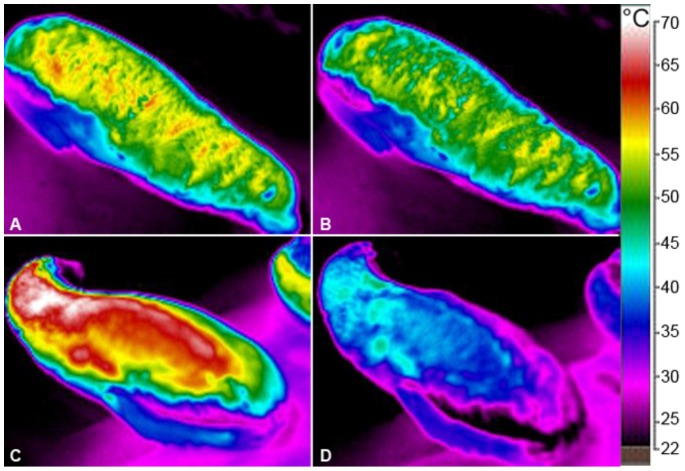
Reduction of T_surface_ on hauled out pups by wind forced convection. Pups hauling out under solar radiation with sudden appearance of a light breeze of wind force 1–2 (Beaufort), corresponding to 0.2–3.3 m/s. (A) Pup with T_max_ = 61.2°C on the surface, T_air_ = 17.3°C. (B) Decreased T_surface_ after light breeze for 5 s to T_max_ = 58.8°C (3.9%). (C) Pup with T_max_ = 72.3°C on the surface, T_air_ = 23.8°C. (D) After 42 s of light breeze T_surface_ decreased to T_max_ = 47.3°C (34.6%).

Although the measurements took place in a wind-sheltered enclosure, there were some situations when a warm light breeze arose during measurements. We observed a mean decrease of maximum T_surface_ of 3.6±0.5% (N = 2) after 5 s and 34.6% (N = 1) after 43 s force 1–2 wind (Beaufort scale), corresponding to 0.2–3.3 m/s. Two examples of wind induced reduction of T_surface_ on hauled out pups at moderate T_air_ are given in Fig. 7. Pups were hauling out in the sun with maximum surface temperature (T_max_) of 61.2°C (7A) and 72.3°C (7C). T_max_ is reduced by 3.9% to 58.8°C after 5 s (7B) and by 34.6% to 47.3°C after 42 s of light breeze (7D), respectively. T_air_ was 17.3°C during the measurements in Figs 7A,B, and 23.8°C during the measurements in Figs 7C,D. On overcast days the pups started shivering and folded the flippers under their bodies as soon as a light wind arose, even with dry fur and at moderate T_air_ between 14°C and 21°C.

## Discussion

### Dry fur

The fur seal pups in our study were exposed to intense solar radiation for extended periods of time, to high air temperatures up to 35.5°C, and fluctuations of air temperature of more than 20°C. They had to cope with temperatures close to 80°C on their fur surfaces. Nevertheless they maintained a stable rectal temperature of 36.9±0.3°C. This is within the range of body temperatures measured in several species of marine mammals from 35.0 to 38.8°C, summarized by Irving [27]. It is also within the range measured in different seal species [12], [13], [25], [28], [29], [30], and can be regarded as normal temperature. Neither high ambient temperatures nor hauling out in the sun for 3 hours caused an increase of T_body_ (pups hauled out in the sun even longer than 3 hours without any attempts of behavioural thermoregulation). On the contrary, our results even indicate a decrease in T_body_ with increased T_surface_ and exposure to solar radiation. This is in contrast with observations in adult California sea lions, that face a significant increase in body temperatures after 80 min staying ashore at air temperatures of 30°C [20]. Furthermore, T_skin_ had a mean value only 1.5°C higher than mean T_body_ as long as the fur was dry. These findings indicate that it is the natal coat that serves as a barrier against solar radiation and high temperatures.

Limberger et al. [25] investigated pups of the Galapagos fur seal, which were also exposed to intense solar radiation. They measured constant core body temperatures of 37.7±0.3°C (mean ±s.d.) as well as skin temperatures up to 3.3°C above core body temperature, slightly higher than our results. Surface temperature on Galapagos fur seal pups exposed to the sun did not exceed 61°C, which is nearly 20°C lower than the maximum T_surface_ in the present study. The duration of exposure to solar radiation is not reported by Limberger et al. [25]; pups used behavioural strategies such as retreat into the readily available shade and wetting in tide pools for thermoregulation. In addition, a steady wind (mean wind speed > = 1 m/s during daytime) prevailed. Therefore the habitat of Galapagos fur seals is totally different from the present study site and the breeding sites of the South African fur seal. Nevertheless, in the present study, the natal fur protected the pups from solar radiation and high temperatures.

The slightly lower mean T_body_ in our results compared to the mean value obtained by Limberger et al. [25] could be caused by the fact that most of our measurements were carried out while the pups were asleep, while core body temperature in the Galapagos fur seal pups was measured during sleep and activity. During sleep body temperature decreases in most mammals, while activity causes an increase in body temperature(e.g. [31]). This has also been described in several seal species [12], [13], [28]. Another reason could be the different method used by Limberger et al. [25], since they used force fed temperature transmitters, enabling measurements in the stomach, which might be closer to the body core than rectal temperature. Nevertheless we consider rectal temperature measurement a suitable method to obtain the core body temperature of otariid pups. Their relatively small body size at birth (54–75 cm standard length measured in Northern fur seals *Callorhinus ursinus* [14], [32]) of which 25–28% account for the pup's head, as well as the pup's thin blubber layer of 2–4 mm [14], [33] allow measurements close to the body core with a thermometer of adequate shaft length. In our study, pups had a trunk length of approximately 20 cm at birth which grew to approximately 35 cm during the study periods. The thermometer was inserted approximately 9 cm (see “Materials and methods”). Even if deep core body temperature had been slightly higher than rectal temperature it is rather improbable that it was more variable than rectal temperature. Therefore rectal temperature was a suitable index for core body temperature variations.

In order to get a more precise insight into the insulating properties of the pups' natal coat we included a further “layer” of temperature measurements between skin and surface into our investigations: in addition to T_body_, T_skin_ and T_surface_, we measured the temperature of the air layer within the pups' fur, an approach that had been proposed by Hammel [34] but has never been applied in live pinnipeds. The regressions in Fig. 4 show a larger temperature difference from the centre of the fur to the surface, meaning the outer 5 to 12 mm of the fur, than to the skin. This result is a little surprising taking the under fur of approximately 5 mm length (Erdsack, unpublished observation) into account. This additional hair, present only in the 5 mm layer close to the skin, would contrarily imply a stronger insulation by the inner part of the fur and therefore a larger temperature difference from the skin to the centre of the fur than between centre and surface. But at this point it must be taken into account that the extremely high temperatures measured on the pups' fur surfaces were caused mainly by solar radiation. We suppose that the curved structure of the upper part of the erected hairs of the pups' coat (see Results, “Natal fur structure”) improves air circulation within the fur and thereby increases convection. Thermal forced convection will be directed upwards, away from the skin. This could be an explanation for the larger temperature difference between centre of fur and fur surface as observed in our study. The exceptional structure of the natal fur has not previously been reported and is currently being investigated in detail. The control measurements indicate that the positions of the temperature measurements within the fur were on average centred between skin and surface (mean position 0.1 mm from the centre, with a standard deviation of 1.2 mm). Therefore we believe that the temperature halfway between the skin and the surface is actually closer to the skin temperature than to the surface temperature, as discernible in Fig. 4. Temperature ranges (>60°C on the surface, >40°C within the fur, 7.2°C on the skin, 1.6°C body core) as well as temperature differences (>40°C between skin and surface, >30°C between fur and surface, >20°C between skin and fur) decrease strongly from surface to body core. This adds evidence that the major part of insulation takes place within the pups' natal coat.

Trites [35] provided theoretical estimates of lethal air temperatures for Northern and Galapagos fur seal pups, which appear to contradict experience and are vastly inconsistent with our measurements. He concluded that with dry fur, in still air and full sunlight, −20°C air temperature would be the upper lethal temperature for Northern fur seal pups in Alaska and −30°C would be the upper lethal temperature in California, while the lower lethal temperature at both sites exceeds −40°C even at night and with wind speeds of 17.6 m/s. For pups of the heat adapted Galapagos fur seal he estimated an upper lethal air temperature of about +5°C at a light breeze (1.17 m/s) in full sunlight, and a lower limit of, again, lower than −40°C, independently of solar radiation. As most of the time there was still air at our study site and air temperatures often exceeded 30°C, we conclude that these lethal limits are not realistic. Though our measurements were conducted in Cape fur seal pups that are neither as cold adapted as the Northern fur seal nor as heat adapted as the Galapagos fur seal, it is rather improbable that the temperature limits between these species differ by more than 50°C. From our observations of the pups shivering by the impact of light wind at temperatures not lower than 15°C we would assume that the lower critical air temperatures between 4 and 0°C, assumed by Blix et al. [33] for newborn Northern fur seal pups, are more realistic.

### Wet fur

Our results show that the dry natal coat protects Cape fur seal pups during their terrestrial stage of life against overheating as well as against hypothermia down to a certain extent. Wetting, by contrast, reduces the insulating power of the natal coat severely. This was obvious by the behaviour of the wet pups (shivering, reducing surface area by folding the flippers under the trunk). Furthermore the wet hair was so flattened and stringy, not containing a persistent air layer any more, that temperature measurements within the fur became nearly impossible. The significantly reduced T_skin_ with wet fur (Fig. 6), which is 1.1°C lower than mean T_body_, is further evidence for the strongly reduced fur insulation. This matches the findings of Blix et al. [33] who assessed the thermal conductance of wet natal fur in Northern fur seal pups to be ten times that of dry natal fur. Furthermore Blix et al. [26] measured a decrease in subcutaneous temperature of up to 20°C on the back of newborn pups wetted by artificial rain. Irving et al. [13] submerged Northern fur seal pups partially in cold water and observed not only a reduction of subcutaneous temperature close to water temperature in the submerged body parts, but even in the dry body parts. This was not the case in adults, which demonstrates very different properties of natal and adult fur. Trites [35], as for pups with dry fur as discussed above, also calculated lethal temperatures for fur seal pups with wet fur. In still air and full sunlight he estimated +5°C air temperature as upper lethal temperature for Northern fur seal pups in Alaska and −5°C in California, respectively, while the lower lethal temperature in still air on clear nights is estimated at −20°C and −25°C, respectively. At 17.6 m/s wind speed lower lethal temperature is estimated at about +5°C at both sites. For Galapagos fur seal pups with wet fur he estimated an upper lethal air temperature of about +25°C at a light breeze (1.17 m/s) in full sunlight and a lower limit at about 5°C on clear nights. Neither the upper nor the lower limits for Northern fur seal pups with wet fur in still air estimated by Trites [35] seem realistic. Our results show that pups are able to cope with much higher air temperatures than 5°C as well as intense solar radiation, even in still air. The lower limits appear very low, especially concerning the fact that below 0°C the water in the fur would freeze, which would prevent the fur from regaining its thick insulating air layer.

We did not find significantly reduced T_body_ when the pups' fur was wet as compared to when the fur was dry, in contrast to former studies, where T_body_ decreased below normal body temperature. On the contrary, Blix et al. [33] observed a continuous decrease of rectal temperature from >37°C down to 34°C within 30 min in a newborn Northern fur seal pup while wetted by artificial rain. Donohue et al. [36] found a correlation between core body temperature and water temperature in submerged Northern fur seal pups before their first moult, but not in post-moult pups, already wearing an adult type fur, which is another indication for the lower insulating power of the wet natal fur.

These findings about the wet natal fur are reminiscent of the properties of the fur of terrestrial mammals. Webb and King [37] for example determined that wetting reduces the total resistance of mammalian fur by 50%. Cuyler and Øritsland [38] estimated a reduction of the insulating power of the fur of the Svalbard reindeer (*Rangifer tarandus platyrhynchus* (Linnaeus, 1758)) by 50% during strong rain. Our findings are in line with the assumption that the natal coat of fur seal pups is an adaptation to the terrestrial life phase of the pups, as it provides an effective barrier against heat and presumably cold, as long as it is dry, but loses much of its insulating power when wet. Similarly the natal fur of ice breeding phocid seals, such as harp seals (*Pagophilus groenlandicus*) and hooded seals (*Cystophora cristata*), that differs strongly in composition, density and hair structure from fur seal natal fur, contributes significantly to the insulation against hypothermia in air, but loses a significant part of its thermal resistance when submerged [2].

By contrast to the effectively insulating fur of adult fur seals, which is useful in the water and at low ambient temperatures but can lead to heat stress on shore at high ambient temperatures [12], [13], [39], the natal fur protects the pups during their sun exposed terrestrial life.

### Impact of wind induced convection

Wind, even a light breeze, has a significant influence on surface temperature and thereby on thermoregulation (see Fig. 7). De Villiers and Roux [39] found a correlation of wind speed and direction with the mortality of South African fur seal pups in Namibia. They discuss that changes in air temperature with wind direction, as they are well known in coastal areas, were responsible for the effect of wind direction. As we observed, even a light breeze of force 1–2 (Beaufort), corresponding to 0.2–3.3 m/s, at moderate air temperatures reduced T_surface_ by 3.6% within 5 s. Heat loss by forced convection depends on wind velocity and air temperature. Therefore a steady onshore wind with high velocities of e.g. 18 m/s, like in the study of De Villiers and Roux [39], bringing cool temperatures from the sea, would reduce surface temperature several fold. Taking the example shown in Fig. 7A with mean T_surface_ of 54.3°C and T_air_ = 17.3°C, and assuming an exposed surface area of 1000 cm^2^ [33] a light breeze of 1.7 m/s would cause a heat dissipation of about 27 W (for method of calculation see [40]). With a strong onshore wind with a velocity of 18 m/s like in the study of De Villiers and Roux [39] and an assumed temperature of 12°C of the seaside wind, heat loss from the back of a pup would increase nearly fourfold to 100 W. These values give an explanation for the increased pup mortality by overheating as a consequence of reduced wind velocities or changes of wind direction, as observed by De Villiers and Roux [39].

### Impact of time of day

Though time of day tended to have an influence on T_body_, the slightly increased T_body_ in the evening is still within the range of normal temperature and therefore presumably not of physiological relevance. Increased T_body_ after 5:00 p.m. probably originated from the fact that all pups were suckled mainly between 4:00 and 5:00 p.m. after the final feeding of the adults. Pups often suckled for up to 1 hour. The increased metabolism may have led to a slight increase in body temperature.

### Conclusion

Our results suggest that the natal coat of fur seal pups, containing an up to 25 mm thick air layer inside the fur, is protective during the pups' terrestrial stage of life. It provides an effective protection against hyperthermia as well as – down to a certain extent – against hypothermia. Insulation against hypothermia is drastically reduced when the fur is wet, in contrast to the adult animals. Pups maintained core body temperature for at least three hours at high air temperature (27°C) in still air (wind speed below 0.19 m/s) under solar radiation that caused the surface of the fur to reach 77°C. Calculations show that wind, if present, would provide significant additional cooling power. A larger portion of the insulating power against heat by radiation resides in the outer than in the inner half of the fur, which is presumably caused by the unique fur structure, enabling air circulation and thereby convection within the outer part of the coat. In a dry state fur seal pups are able to maintain a constant core body temperature independent of environmental parameters as well as a relatively stable skin temperature close to body temperature, despite high surface and ambient temperatures and intense solar radiation for hours. We believe that the natal fur is specifically adapted to protect the pup against heat, while also providing significant insulation against cold; these two functions appear in part to be divided between the outer and the inner half of the fur. Morphological investigations of the fur to investigate the mechanisms of heat insulation will significantly add to our knowledge on this topic and are being conducted.
